# Multifunctional Polymer Nanocomposites Reinforced by Aligned Carbon Nanomaterials

**DOI:** 10.3390/polym10050542

**Published:** 2018-05-17

**Authors:** Shuying Wu, Shuhua Peng, Chun H. Wang

**Affiliations:** School of Mechanical and Manufacturing Engineering, University of New South Wales, Sydney NSW 2052, Australia; shuying.wu@unsw.edu.au (S.W.); shuhua.peng@unsw.edu.au (S.P.)

**Keywords:** multifunctional, polymer nanocomposites, alignment, carbon nanomaterials

## Abstract

Carbon nanomaterials such as carbon black (CB), carbon nanotubes (CNTs), and graphene have demonstrated significant potential as fillers to improve the electrical, thermal, and mechanical properties of polymers and their fiber-reinforced polymer composites. The level of improvement has been found to depend significantly on the degree of alignment of carbon nanomaterials. Due to the very small scale and complex interactions of carbon nanomaterials with polymers and structural fibers, alignment in a given direction has been a major challenge. Over the past decade, considerable effort has been devoted to developing effective strategies to align carbon nanomaterials in polymer matrices. However, significant technological challenges remain, and there is still a lack of understanding of the alignment mechanisms and their effects on the properties of polymers and composites. This paper reviews in situ alignment techniques including shear deformation, mechanical stretching, electrospinning, and application of an external magnetic or electric field, and ex situ techniques including using vertically grown CNTs or graphene. This review particularly focuses on physical mechanisms underpinning the magnetic or electric field-induced alignment and theoretical analyses that describe the different motions occurring and the major parameters controlling alignment. Moreover, this review highlights the recent research findings of the effects of alignment on the properties of polymer nanocomposites. The outlook towards the challenges and opportunities in this field are also discussed in this review.

## 1. Introduction

Polymer nanocomposites are one type of organic-inorganic hybrid materials where inorganic nanoscale fillers, such as nanoparticles, nanotubes, or nanosheets, are dispersed in an organic polymer matrix. Compared to conventional fiber-reinforced polymer composites (FRPs), where the reinforcement fibers are on the micrometer scale, polymer nanocomposites are characterized as composites with constituents on the nanometer scale. Polymer nanocomposites offer several major advantages, including much greater interface area between fillers and matrix. As a result, nano-scale fillers can create remarkable improvements in composite properties, even at low concentrations (typically less than 5 vol %) so that the polymers’ processability are not adversely affected. It is widely recognized that there is a trade-off between desired performance, mechanical properties, cost, and processability. The much lower concentration of fillers enables greater retention of the inherent processability of neat polymers [1].

Nanocomposites are now widely used in various applications including packaging, automotive, aerospace, defense, electronics, semiconductors, coatings, energy, sports, medical, and healthcare. The total global market for polymer nanocomposites is expected to reach above $5 billion by 2020, growing at a faster annual growth rate than that of conventional polymers [2]. The most commonly used nanofillers include nanoclay, graphene nanoplatelets (GNPs), carbon nanotubes (CNTs), carbon black (CB), and carbon nanofibers (CNFs). The incorporation of these nanofillers into polymers can greatly improve the mechanical properties (tensile strength, stiffness, fracture toughness, etc.), enhance thermal stability and flame-retardant properties, and decrease gas permeability. Carbon nanomaterials (such as CB, CNTs, graphene, and CNFs) have drawn special attention in the development of multifunctional composite materials, which is most likely due to their excellent mechanical, thermal, and electrical properties.

Polymer/carbon nanomaterials composites exhibit greatly improved mechanical properties due to the strong interactions with the matrix, resulting from the nanoscale structure and very large interfacial area [3]. Experimental investigation of the elastic modulus and electrical conductivity of graphene-based [4,5] and/or CNTs-based [6,7] polymer nanocomposites demonstrates that the interfacial average shear stress of polymer/CNTs composites is in the order of 500 MPa, which is much larger than that exists in conventional fiber composites. The results also revealed that the elastic modulus of the nanocomposites is generally improved while their ultimate strength and strain to failure are often degraded. Moreover, carbon nanomaterials have proven to be very effective as conductive fillers due to their high electrical conductivity [8]. Two-dimensional graphene can also improve significantly the gas barrier properties of polymers [9]. The superior properties of carbon nanomaterials offer exciting opportunities for new composites.

However, the property improvements by carbon nanomaterials achieved thus far are much lower than the theoretical predictions. Critical challenges to maximizing property improvements include the dispersion, exfoliation, and alignment of nanofillers. There is a large volume of literature addressing the dispersion issue [2,10,11,12,13,14,15,16,17,18,19,20]. Carbon nanomaterials tend to agglomerate due to Van der Waals forces between particles. To improve their dispersion, various methods have been developed, including chemical functionalization [14], noncovalent functionalization [2,12], in situ polymerization [16], and high power dispersion methods such as ultrasound and high speed shearing [17,18,19,20]. These approaches result in uniform dispersion, but carbon nanomaterials are often randomly oriented in the polymers.

The alignment of carbon nanomaterials (CNTs) in polymers was firstly introduced by Ajayan et al. [21], who demonstrated that a cutting process can induce anisotropy in epoxy/CNTs nanocomposites. Their research indicated how to organize nanotubes into well-aligned arrays on the micrometer scale, and conceptually revealed that the nanotube properties can be practically transferred to polymers. Since then, this topic has received much attention and various approaches have been developed to align carbon nanomaterials in polymers to attain the desired composite properties. In general, the alignment of carbon nanomaterials in a polymer matrix can be achieved: (1) “In situ”, i.e., alignment occurs during the formation of composite or (2) “ex situ”, i.e., carbon nanomaterials are aligned in advance, then incorporated into polymer matrices to form composites. Despite these recent advancements, the most commonly used composite preparation methods, such as solutions or melt blending, can compromise the alignment of carbon nanomaterials. Moreover, some well-aligned carbon nanomaterials tend to revert to random orientation upon their incorporation into polymer matrices. Therefore, practicable alignment techniques that are applicable to different polymer matrices remain a challenge.

In contrast to the dispersion issues that have been widely reported, few reviews are available in the literature which present the directional alignment of carbon nanomaterials in a polymer matrix [3,22,23,24]. To the authors’ knowledge, Xie et al. [3] are the first researchers who present a brief review of the techniques for aligning CNTs in polymers, without discussing the alignment mechanisms. Ahir et al. [22] reviewed various techniques to disperse nanotubes in a polymer matrix with a short discussion on effects of alignment on the photo-stimulated mechanical response of polymer nanocomposites. More recently, Sun et al. [24] highlighted new synthetic methods for aligning continuous CNT sheets and fibers for applications in organic optoelectronic devices. Another recent review by Goh et al. [23] presents contemporary alignment approaches and compares advantages and disadvantages of the different approaches. However, these review articles have not addressed a number of major issues pertinent to the alignment of carbon nanomaterials: (a) the underlying alignment mechanisms; (b) theoretical modeling of electric or magnetic field-induced alignment of carbon nanomaterials, including not only the carbon nanotubes, but also some other carbon nanomaterials such as graphene and carbon nanofibers; (c) quantitative evaluation of the impact of alignment of carbon nanomaterials on the mechanical, electrical, and thermal properties of their polymer nanocomposites. The reinforcing mechanisms, especially toughening mechanisms induced by aligned carbon nanomaterials, have rarely been reported. This review firstly introduces the alignment techniques developed to date, including “ex situ” and “in situ” methods. Among the “in situ” techniques, we place our attention on the theoretical modeling and recent experimental investigations of the electric or magnetic field-induced alignment of carbon nanomaterials in a polymer matrix. This review highlights the effects of alignment on the mechanical, electrical, and thermal properties of polymer nanocomposites. The underlying mechanisms responsible for these influences are also discussed. This review provides a general guide for feasible processing methods for reinforcing polymer nanocomposites by aligned nanomaterials.

## 2. Alignment Techniques

### 2.1. Overview of Different Alignment Techniques

To fully take advantage of the excellent properties of individual carbon nanofillers, it is desired that the nanofillers are aligned in the polymer matrices. It is commonly recognized that mechanical properties such as stiffness, strength, and fracture toughness, and functional properties such as electrical, thermal conductivities, and piezoresistivity of a nanocomposite depend strongly on the orientation of the nanofillers in the matrix, very similar to conventional fiber-reinforced polymer composites. Most of the established alignment methods involve using external forces such as electric or magnetic fields, shear force, or mechanical stretching. The alignment can be achieved using “ex situ” or “in situ” techniques before or after the incorporation of polymers.

#### 2.1.1. “Ex Situ” Alignment

**Figure 1:**
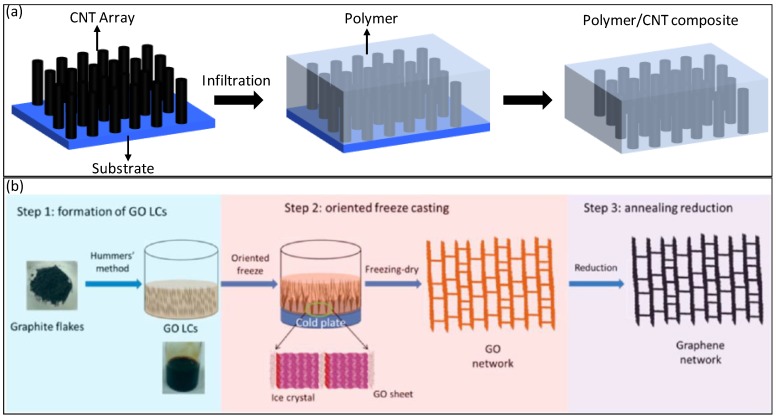
(**a**) Schematic illustration of the fabrication of vertically aligned carbon nanotubes/polymer composites; (**b**) schematic illustration of the formation of vertically aligned and interconnected reduced graphene oxide networks [29]. Reproduced with permission. Copyright American Chemical Society, 2016.

Well-aligned carbon nanomaterials can be fabricated and then compounded with polymers to develop nanocomposites. This method is challenging as it requires the preparation of nanomaterials arrays with a well-aligned and robust structure in the first place. Polymers can then be infiltrated into the arrays, as indicated in Figure 1a. This fabrication process has been applied to vertically aligned CNTs and their polymer composite thin films [25,26]. Vertical aligned CNTs and CNFs have been developed through different techniques including plasma-enhanced chemical vapor deposition (CVD) [27] or the conventional CVD method [25,26,28]. Recently, vertical reduced graphene oxide (rGO) has been developed by directional freeze casting followed by high temperature annealing (Figure 1b) [29]. Another challenge associated with the “ex situ” alignment method is that the polymer infiltration process may alter the structure of the aligned nanomaterials. Combining Euler beam theory and viscous drag and the capillary force model, Buchheim et al. [28] demonstrated that polymers exert a capillarity-induced attractive force laterally on vertically-aligned CNTs, which results in locally agglomerated macroscale bunches. The highly aligned structure is destroyed as an individual CNT is buckled and distorted.

#### 2.1.2. “In Situ” Alignment

The alignment of carbon nanomaterials in a polymer matrix can also be performed during the formation of the composites. Commonly used techniques include the application of shear force, mechanical stretch, magnetic field, and electric field. Electrospinning has also been demonstrated as an effective way to align carbon nanomaterials. This section briefly introduces these techniques.

Two commonly used methods to manufacture polymer nanocomposites are resin infusion and injection molding. The suspended nanofillers (mostly CNTs) inside the polymer matrix change their orientation due to the flow-induced shearing, which has been investigated experimentally and theoretically [30,31,32,33,34,35,36,37,38,39,40,41]. The transfer of shear force from the polymer matrix to the CNTs leads to their deagglomeration and orientation along the flow direction. The shear rate, nanotube concentration, aspect ratio, and total shear play crucial roles in determining the CNT network structure. Eken et al. [34,36] found that high shear rates facilitate the deagglomeration of CNTs and the orientation along the flow direction. At low shear rates, however, the motion of the nanotubes causes a build-up of agglomerates and a low degree of alignment. Eken et al. used fiber-level simulations to study the combined effect of the CNT aspect ratio and shear rate and found that at a low aspect ratio nanotube dispersions exhibit higher anisotropy than the high aspect ratio nanotube dispersions. Similarly, Sulong et al. [37] and Fan at al. [40] both reported that the degree of CNT alignment increases with increasing shear rate. Another important question is whether the orientation of CNTs changes to random after the shear is discontinued and before the suspension solidifies. Fan at al. [40] used an order-of-magnitude analysis to estimate the Brownian motion effect theoretically and found that the Brownian motion has a negligible effect on CNT orientation, and little or no CNT alignment relaxation was observed after the shear force was discontinued.

**Figure 2:**
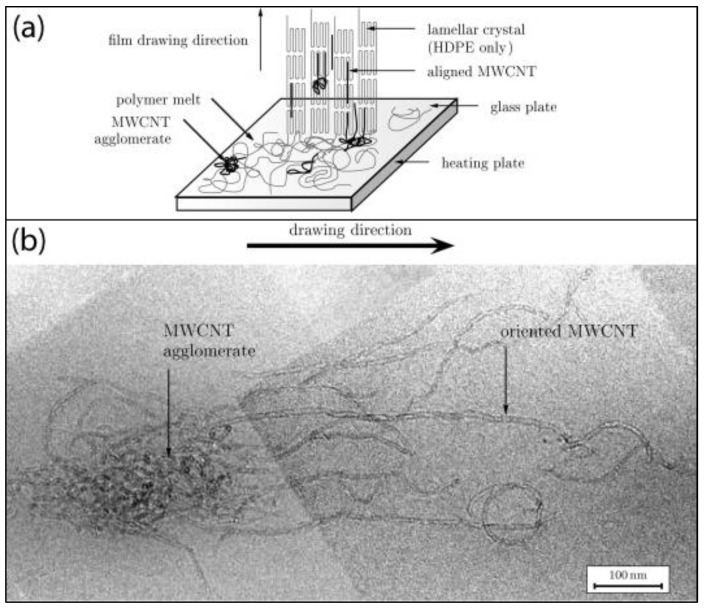
(**a**) Schematic melt-drawing of a multiwall carbon nanotubes/high-density polyethylene (MWCNTs/HDPE) composite film; (**b**) a representative TEM micrograph of MWCNTs/isotactic polypropylene melt-drawn film which indicates that the MWCNTs are disentangled and aligned by the drawn polymer [44]. Reproduced with permission. Copyright Elsevier Ltd., 2013.

Mechanical stretching is a versatile technique to induce strong molecular orientation and thereby crystallization in semi-crystalline polymers. Therefore, it has been used to promote the alignment of nanofillers in their polymer composites [42,43,44]. A recent study by Arras et al. [44] indicates that CNT alignment and disentanglement can be induced by melt-drawing. As illustrated in Figure 2a, high-density polyethylene (HDPE) forms staggered lamellar chain-folded crystals while the multiwall carbon nanotube (MWCNT) agglomerates, which are dispersed in the HDPE melt, are disentangled and aligned during the melt-drawing. A representative Transmission Electron Microscopy (TEM) image (Figure 2b) reveals that the disentangled MWCNTs were pulled-out in the drawing direction. An exceptionally high degree of local MWCNT alignment parallel to the films’ drawing direction was achieved, with an angular deviation of <10° (in HDPE) and <20° (in isotactic polypropylene or poly(1-butene)). Another study by Zhang et al. [42] demonstrated that melt-spun single-walled carbon nanotubes (SWCNTs)/poly(l-Lactide) (PLLA) composite fibers showed a great improvement in tensile strength and elongation at break than neat PLLA when prepared under a high draw ratio (take-up speed 50 m·min^−1^). Both the PLLA chains and SWCNTs were well oriented along the draw direction. The PLLA chains nucleate and grow on the SWCNTs surface, forming an interfacial brush-like hybrid structure. The remarkable reinforcement effect is mainly ascribed to the interfacial crystallization formed during the stretching process.

Another widely used method for fabricating polymer composites with aligned carbon nanomaterials is electrospinning, which employs an applied electric force to draw out fibers from the tip of a sharp conical meniscus. Dror and coworkers [45,46] were one of the pioneers who explored aligning CNTs using the electrospinning technique in a polymer matrix. A theoretical model was proposed, mimicking the behavior of rod-like particles (representative of CNTs) in electrospinning. The rods were initially randomly oriented and gradually aligned along the stream lines due to sink-like flow in the cone, and consequently, a high degree of alignment in the electrospun fibers. It was found that the alignment of CNTs depends on the quality of the initial dispersion, polymer matrix, and the interaction between spun polymer and CNTs as well as the wettability [45,47].

Applying an external field, i.e., an electric field or magnetic field, has been considered as one of the most efficient and straightforward approaches to assist the assembly of fillers in a polymer matrix to drastically improve its directional properties [23]. The use of external fields to manipulate the properties of materials originated from electrorheological and magnetorheological fluids [48]. These fluids consist of suspensions of micrometer size particles in liquid mediums. When subjected to an external electric field or magnetic field, the particulate particles are polarized, generating electric dipoles or magnetic dipoles. The interaction of opposite dipoles results in the chaining of particles. The mechanisms of the alignment will be discussed in the following section together with some experimental examples.

### 2.2. Electric Field-Induced Alignment: Mechanisms and Modeling

The electric field-induced reorientation was first demonstrated by Yamamoto et al. [49,50] who revealed that CNTs can be rotated to the electric field direction in isopropyl alcohol using direct current (DC) and/or alternating current (AC) electrophoresis for the purpose of purification and individual handling of nanotubes. The degree of alignment of nanotubes increases with increasing frequency from 10 Hz to 10 MHz and the length of nanotubes. The anisotropy of electrophoresis velocity was considered as the reason for alignment of CNTs. Since this early work, electric fields have been employed to induce preferential agglomeration of CB and CNFs to develop polymer composites with anisotropic properties [51,52]. Electric fields can be applied with various configurations of electrodes including parallel plates, interdigitated system of electrodes, or configurations of electrodes producing a nonuniform field.

However, the physical mechanisms enabling the electric field-induced alignment were not well understood until the work by Kim and coworkers’ [53], which identified the prevalent physical mechanisms influencing the formation of the structure of inclusions in an electric field. When subjected to an electric field (E), the double-layer surrounding an inclusion in a dielectric liquid is polarized and acquires a dipole moment, µ [53]:

(1) $$µ=\varepsilon_{0}\cdot \varepsilon_{m}\cdot \beta_{F}\;\cdot V\;$$

where *V* is the volume of the inclusion, $\varepsilon_{0}$ is the free-space permittivity, and $\varepsilon_{m}$ is the relative dielectric constant of the surrounding matrix. The dimensionless parameter $\beta_{F}$ combines the effects of polarizability of the inclusion and the depolarization effect of the inclusion shape. The detailed calculation of $\beta_{F}$ can be found in a previous work [53]. Therefore, a torque $T^{e}=\vec{\mu }\times \vec{E}$ may be induced to act on the inclusion. Carbon nanomaterials including CNTs and graphene are nonspherical in shape and hence a nonzero torque is generated as the major axis of the particles do not align with the electrical field direction. The overall torque acting on the inclusion is the superposition of the torques induced by the fields parallel and perpendicular to their axes $T^{e}=\mu_{\|}\times E_{⊥}-\mu_{⊥}\times E_{\|}$ (where *E*_⊥_ = E·sinθ and $E_{\|}$ = E·cosθ with θ being the angle between the electric field direction and the semi-major axis of the inclusion).

Considering an inclusion moving in a liquid polymer, the resisting torque is due to the viscous drag by the surrounding liquid polymer which is given by Stoke’s law [54]:

(2) $$T^{v}=3\pi \cdot \eta \cdot \nu \cdot d\cdot k^{v}$$

where η is the polymer viscosity, ν is the velocity, *d* is the major diameter of a particle, and $k^{v}$ is associated with the shape of the inclusion which can be found in a previous work [53]. Similarly, a viscous torque acting on a particle rotating in viscous matrix is:

(3) $$T^{v}=6\cdot \eta \cdot V\cdot \;k^{v}\;\left(\frac{1}{2}\mathrm{curl}\left(\nu_{\infty }\right)-\;\dot{\theta }\right)$$

where $k^{v}$ is a coefficient relating to the shape of the inclusions and $\dot{\theta }$ is the angular velocity. The dynamics of the inclusion are determined by balancing the driving force induced by the electric field and viscous interaction with the polymer matrix. The equation can be used for estimating the time required to translate or rotate an inclusion of any shape and configuration.

**Figure 3:**
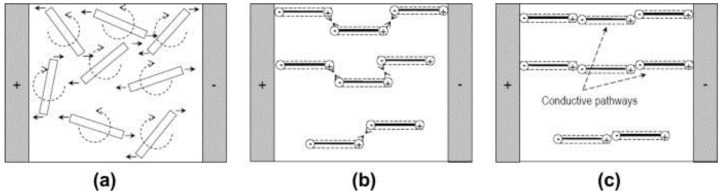
Mechanism of the CNT conductive network formation: (**a**) Orientation to the electric field direction; (**b**) end-to-end attraction of polarized CNTs; (**c**) formation of the highly oriented CNT network [55]. Reproduced with permission. Copyright Elsevier Ltd., 2012.

Monti et al. [55] recently investigated the alignment of SWCNTs in an epoxy resin using a DC electric field. A physical modeling was proposed based on electrodynamics describing all the phenomena when subjected to a DC electric field, including the rotation of CNTs, translation of polarized CNTs toward each other to form chain-like structure, and the relaxation mechanism when the electric field is switched off. Figure 3 illustrates the mechanisms for the formation of the CNTs conductive network under electric field. Based on the modeling, the time required for rotating a CNT from an initial angle $\theta_{0}$ to a new angular position θ′ can be estimated by:

(4) $$t\left(\theta^{\prime }\right)=\frac{1}{2A}\ln\frac{\tan\theta_{0}}{\tan\theta^{\prime }}$$

where $A=2\pi \cdot \varepsilon_{0\;}\cdot \alpha_{\left|\right|}\cdot E^{2}/\left(\eta k_{r}\right)$, $k_{r}$ represents the rotational friction coefficient and $\alpha_{\|}$ is the longitudinal polarizability while the transverse polarizability is neglected due to the fact that it is more than one order of magnitude lower than longitudinal polarizability [55,56].

Meanwhile, the polarized nanotubes tend to attract each other due to the opposite charges present at their ends. The interparticle distance x(t) is expressed as $x\left(t\right)=\sqrt[{3}]{3Ct+x_{0}^{3}}$, where $C=\;\frac{4{\pi \varepsilon }_{0}\alpha^{2}E^{2}}{\varepsilon_{r}l^{2}\eta k_{\mathrm{tr}}}$, $k_{\mathrm{tr}}$ is the translation friction coefficient and $x_{0}$ represents the initial distance between two oppositely charged nanotubes. Therefore, the time needed to form the end-to-end CNT connection ($t_{\mathrm{hth}}$) can be written as:

(5) $$t_{\mathrm{hth}}=\;\frac{x_{0}^{3}}{3C}$$

In addition, another important phenomenon that should be considered is the Brownian diffusion caused by random Brownian forces. The orientation and chain-like structure of CNTs are prone to relax and return to their random orientation. The Brownian forces are usually neglected and considered to be much weaker than the electric field-induced forces. However, when the electric field is switched off, the relaxation takes place and Brownian diffusion becomes a dominant factor causing the deconstruction of the network. A previous work [55] presents the equations for calculating how much the CNTs diffuse during time *t*, indicating the relaxation is affected by the temperature, shape (nanotube diameter, length), and viscosity of the liquid medium.

It is now recognized that lower viscosity, higher field strength, and a larger difference in the dielectric constants of the inclusions and liquid medium lead to a higher degree of alignment [17,53,55,57,58,59,60]. The characteristics of the inclusions also play key roles in the electric field-induced alignment, especially the functional groups on the particle surface, which influence the interaction with the polymer matrix [60,61,62]. For instance, the oxidization of CNTs [60,61,62] introduces hydroxyl and carboxylic groups onto the surface, which not only enhances the dispersion quality in polymer matrix, but also improves the alignment quality by preventing the formation of thick bundles. The enhanced interaction with the polymer matrix tends to slow down the alignment process. However, Lim and coworkers [63] demonstrated that the alignment of functionalized CNFs is independent of the functional groups (carboxylic acid or amine groups). Unfortunately, the alignment of the pristine CNFs without functional groups is not investigated in their work. Our most recent work on the alignment of short carbon fiber and graphene nanoplatelets [17,64] indicates that the time required to rotate or translate fibers or graphene is independent of their aspect ratios, consistent with that reported by Kim et al. [53].

**Figure 4:**
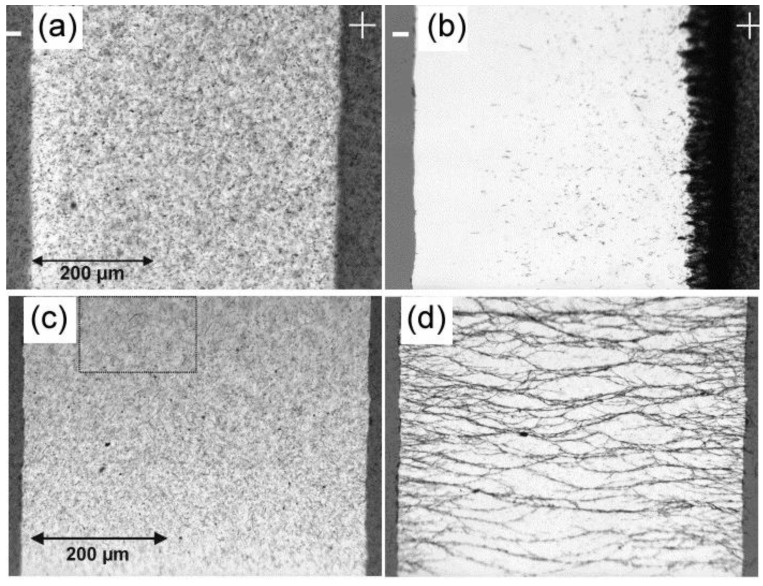
Electrophoresis of 0.2% carbon nanofibers (CNF) in amine curing agent (**a**) before and (**b**) 17 min after the application of DC electric field of 800 V/cm; alignment of 0.2% CNF in amine curing agent (**c**) before and (**d**) 30 min after the application of an AC electric field 400 V/cm and 1 kHz [52]. Reproduced with permission. Copyright Elsevier Ltd., 2003.

Apart from the rotation, relative translation between two oppositely charged particles, and relaxation, the translation motion toward the electrodes may also occur, depending on the external field type and surface charge on the particles. In the case of a DC electric field, CNTs move toward electrodes depending on their electrophoretic mobility. When subjected to an AC electric field, the net electrophoretic mobility equals zero. However, the aligned CNTs cause inhomogeneities in the electric field, leading to the movement of induced dipoles towards the location with the highest field strength. Therefore, CNTs may move towards the closest electrode [65]. Prasse et al. [52] compared the response of CNFs in an epoxy resin under DC and AC electric fields and found that most CNFs were deposited onto the anode under DC field while chain-like alignment was observed under the sine wave AC electric field (as indicated in Figure 4). Similar results were reported by Oliva-Aviles et al. [58] on investigation of MWCNT alignment in dielectric liquid media.

**Figure 5:**
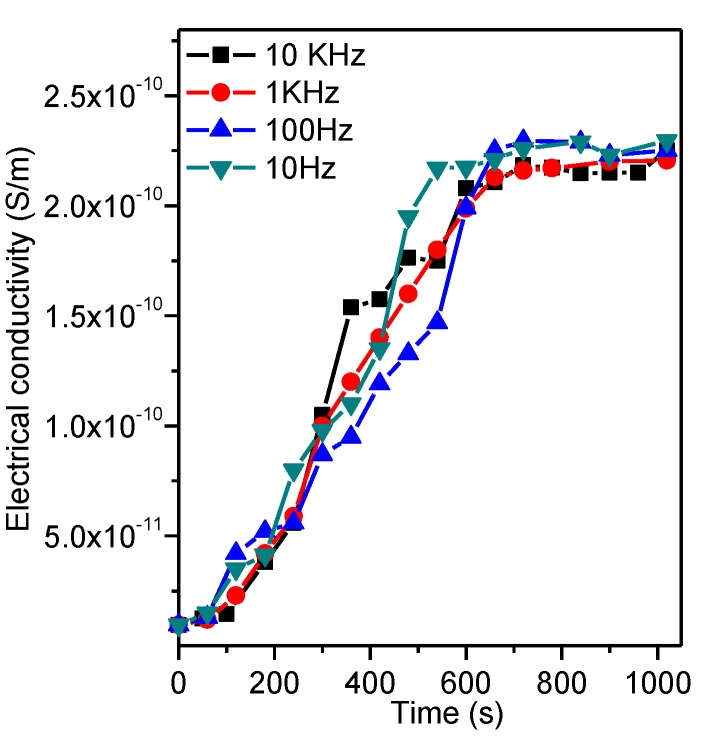
Time dependence of the DC electrical conductivity for the liquid epoxy resin/ graphene nanoplatelets (GNPs) mixture during the application of an AC electric field [17]. Reproduced with permission. Copyright Elsevier Ltd., 2015.

The influence of electric field frequency on alignment was investigated [50,57,58]. In particular, Oliva-Aviles et al. [58] demonstrated that the aligned MWCNT network was formed faster in dissolved polysulfone upon increasing the electric field frequency (in the order of kHz). Yamamoto et al. [50] and Chen et al. [57] found that the degree of orientation of nanotubes increased with frequency (in the range of 10 Hz to 10 MHz). Our recent work [17], however, showed that the frequency dependency of AC electric field-induced alignment of graphene nanoplatelets (GNPs) in epoxy diminishes rapidly as the frequency exceeds $1/t_{r,\infty }$, where $t_{r,\infty }$ denotes the time taken to rotate a graphene nanoplatelet, using an AC field of infinite frequency. The time needed to rotate a GNP from an initial angle θ_0_ to a new angular position θ′ can be calculated by:

(6) $$t_{r}-\frac{1}{2\omega }\sin2\omega t_{r}=\frac{1}{A}\ln\frac{\tan\theta_{0}}{\tan\theta^{\prime }}$$

where ω denotes the angular frequency, $A\;=\;\frac{\pi }{8\eta }\frac{\varepsilon_{m\;}}{\left(\frac{\pi }{2}-\frac{b}{a}\right)}E_{0}^{2}$ in which $\varepsilon_{m}$, η, *a*, and *b* are dielectric constant of the matrix, viscosity of the matrix, semi-major, and semi-minor axes of GNPs (treated as thin oblate spheroids), respectively. The distance between nano-carbon particles, x, after applying the electric field for a period of time *t* can be determined by solving the equation:

(7) $$x\left(t\right)=\sqrt[{3}]{-3B\left(\frac{t}{2}-\frac{\sin2\omega t}{4\omega }\right)+x_{0}^{3}}$$

where $B=\frac{4\pi a^{4}}{9\eta k_{t}\varepsilon_{0}}\frac{E_{0}^{2}\varepsilon_{m}^{2}{}_{\;}}{{\left(\frac{\pi }{2}-\frac{b}{a}\right)}^{2}}$, in which $k_{t}$ is the translational friction coefficient for an oblate spheroid particle, *E*_0_ denotes the amplitude of the applied electric field. The electrical conductivity was measured to monitor the alignment degree. The results indicate that the electrical conductivity increases due to the formation of a graphene network and reaches a plateau after around 600 s irrespective of the frequency of the electric field, as shown in Figure 5 [17]. Similar results have been reported for the epoxy/CB composites and the time-dependent evolution of the conductivities and final conductivity are almost independent of frequency in the range of 100 Hz up to 10 kHz [51].

### 2.3. Magnetic Field-Induced Alignment: Mechanisms and Modeling

The rotation of nanomaterials in magnetic fields has been demonstrated for paramagnetic [66] and diamagnetic particles [67]. This approach has not been extended to the alignment of nanotubes until a publication in 2001 by Fujiwara et al. [68] who revealed that arc-grown MWCNTs were oriented parallel to the magnetic fields when their suspension in organic solvents was paced in a magnetic field of 7 T. The magnetic-field induced rotation arises from the anisotropic susceptibility, i.e., there exists large differences between the diamagnetic susceptibilities parallel ($\chi_{\|}$) and perpendicular ($\chi_{⊥}$) to the particle axis. The magnetic energy of nanotubes is expressed by [68]:

(8) $$E\left(\theta ,H\right)=-\left(nH^{2}/2\right)\;[\chi_{⊥}+\left(\chi_{\|}-\chi_{⊥}\right)\cos^{2}\theta ]$$

where n is the mole number of carbon atoms, H is the field strength, and θ is the angle between the particle axis and the magnetic field H. If $\left|\chi_{⊥}\right|$ is larger than $\left|\chi_{\|}\right|$, the energy E(θ,H) is at a minimum and magnetic orientation occurs. The anisotropic susceptibilities of nanotubes were calculated theoretically and the susceptibility perpendicular to the tube axis was found to be three orders of magnitude larger than that in the parallel direction [69]. Therefore, magnetic rotation takes place.

By using this approach, Kimura et al., for the first time, fabricated electrically and mechanically anisotropic nanotube-polymer composites [70]. The MWCNTs were dispersed in a monomer solution of unsaturated polyester and placed in a magnetic field of 10 T. The in situ polymerization under a magnetic field freezes the position and orientation of the CNTs. In subsequent research, the magnetic field was applied to process CNT or CNF polymer composites to achieve anisotropic properties [71,72,73,74,75]. For instance, Choi et al. [71] applied a 25 T magnetic field to align MWCNTs in epoxy and the resultant epoxy nanocomposites show a 10% and 35% increase in thermal and electrical conductivity along the magnetic field direction. Recently, another similar attempt was reported by Mahfuz et al. [73] who used a strong magnetic field of 28 T to align CNFs in epoxy. Increases of 21% and 3% in compressive strength and modulus were achieved, compared to epoxies containing randomly-oriented CNFs.

**Figure 6:**
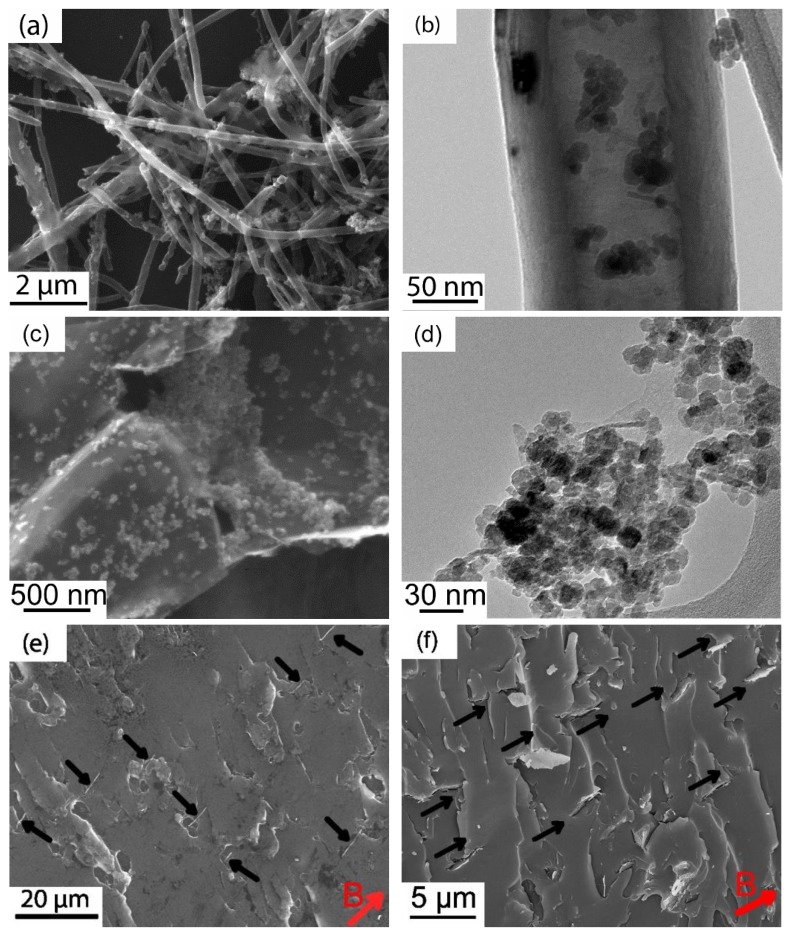
(**a**) SEM and (**b**) TEM images of Fe_3_O_4_/CNF nanohybrids and (**e**) SEM showing its alignment in epoxy composites [84]; (**c**) SEM and (**d**) TEM images of Fe_3_O_4_/GNP nanohybrids and (**f**) SEM image showing magnetic field-induced alignment of Fe_3_O_4_/GNPs [20]. Reproduced with permission. Copyright Elsevier Ltd., 2015 & 2016.

Despite the promising results, the need to apply very high magnetic fields (usually several T) for alignment largely limits the practical applications of this technique, due to the low magnetic susceptibility of carbon nanomaterials. Therefore, various approaches have been reported to functionalize carbon nanofillers with magnetic nanoparticles so that they can be aligned by a lower magnetic field [20,76,77,78,79,80,81,82,83,84,85,86]. For instance, the pristine CNTs synthesized by CVD contains residual catalyst nickel particles which enables alignment by a low magnetic field of 0.4 T [83]. Alternatively, magnetic nanoparticles such as magnetite/maghemite nanoparticles [20,76,77,79,80,81,82,84,85,86] or ferromagnetic nickel and cobalt nanoparticles [78] have been attached onto the surface of the CNTs or graphene. The coating of magnetic nanoparticles greatly increases the magnetic susceptibility, facilitating alignment under low magnetic fields (normally less than 1 T field is required). For instance, the present authors recently coated CNFs and/or GNPs with magnetite nanoparticles (Figure 6a–d) and found that such magnetic nanohybrids can be aligned by a magnetic field as low as 9 mT [20,84], as indicated by Figure 6e,f.

**Figure 7:**
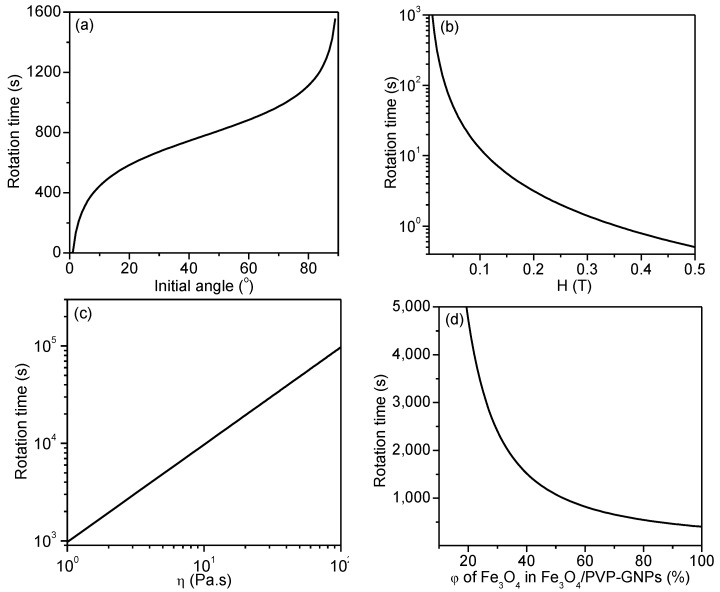
Plots indicating the dependence of the rotation time (*y*-axis) on (**a**) initial angle for the Fe_3_O_4_/GNPs nanohybrids; (**b**) magnetic field strength, *H*; (**c**) viscosity of the suspension, η; and (**d**) volume fraction of Fe_3_O_4_ in the Fe_3_O_4_/GNPs, φ [20]. Reproduced with permission. Copyright Elsevier Ltd., 2016.

The magnetic field-induced alignment of GNPs coated with magnetite nanoparticles in liquid epoxy resin was theoretically analyzed [20]. When exposed to an external magnetic field, Fe_3_O_4_ nanoparticles generate magnetic moments acting on GNPs thus forcing the Fe_3_O_4_/GNPs nanohybrids to align to the direction of the magnetic field. By solving the equation of dynamic rotation, the time ($t_{r}$) to rotate a Fe_3_O_4_/GNPs from an initial angle of $\theta_{0}$ to a generic angular $\theta^{\prime }$ can be calculated by:

(9) $$t_{r}=\frac{1}{2A}\ln\frac{\tan\theta_{1}}{\tan\theta_{2}}$$

where θ represents the angle between the magnetic field vector and the platelet’s long axis, $A=\frac{{\pi \mu }_{0}\chi_{nh}^{2}}{16\eta \left(\chi_{nh}+1\right)a^{3}}\left[\left(b+d\right){\left(a+d\right)}^{2}-ba^{2}\right]H^{2}$ in which $\mu_{0}$ is the magnetic permeability of free space, *d* represents the diameter of the Fe_3_O_4_ nanoparticles, *H* is the external magnetic field strength, *χ_nh_* is the magnetic susceptibility of the Fe_3_O_4_/GNPs, which is a function of the magnetic susceptibility of the Fe_3_O_4_ nanoparticles and their volume fraction, φ, η is the viscosity of the fluid medium, and *a* and *b* represent semi-major and semi-minor axes of GNPs, which are treated as thin oblate spheroids. Figure 7a shows the time required for rotating a Fe_3_O_4_/GNPs from various initial angles to within 1.0° off the magnetic field with a strength of 9 mT. Figure 7b demonstrates that the rotation time drops significantly upon increasing the strength of magnetic field up to 0.04 T, above which the dependency on strength is less noticeable. Figure 7c,d indicate that the rotation time decreases linearly as the viscosity of the suspension increases, while a dramatic decrease is seen upon increasing the volume fraction of Fe_3_O_4_ in Fe_3_O_4_/GNPs.

## 3. Properties of Polymer Nanocomposites Containing Aligned Carbon Nanofillers

Due to their unique structure and remarkable mechanical, electrical, and thermal properties, carbon nanomaterials have been widely studied as a second phase to produce high performance polymer composites. The incorporation of carbon nanomaterials improves the functionalities of polymers and greatly expands their applications. Carbon nanomaterials, such as CNTs and graphene, typically provide high mechanical strength, high electrical and thermal conductivities, and excellent thermal stability. However, the properties of composites containing randomly oriented nanomaterials are much lower than what could be achieved if their nanomaterials were aligned [87]. Some attempts have been made recently to improve the properties of polymer nanocomposites by aligning the nanofillers. The alignment facilitates the formation of interconnecting network for transferring phonons and electrons, thereby improving electrical and thermal conductivities. Given that the laminated FRPs exhibit very different in-plane and through-the-thickness mechanical properties, the alignment of nano-reinforcements may also provide anisotropic mechanical performance. This section highlights the anisotropic properties of polymer composites containing aligned carbon nanomaterials. 

### 3.1. Electrical Properties

Recently, the addition of electrically conductive carbon nanomaterials especially the 1D CNTs and CNFs, and 2D graphene with high aspect ratios to form conductive nanocomposites, has stimulated a surge of scientific interests. These conductive nanocomposites are finding their applications in aerospace for dissipating electrostatic charges [88], strain/damage sensors for health monitoring [89,90,91], stretchable electronics [92], and electromagnetic interference (EMI) shielding materials [93], to name a few. Even when dispersing very small amounts of these nanomaterials, the electrical conductivity of the insulating polymers can be significantly enhanced. Two electrical conductivity mechanisms, electron hopping (quantum tunneling) at the nanoscale and conductive networks at microscale, have been recognized as the main conducting mechanisms for percolation behaviors [94]. At extremely low concentrations, the interparticle distance is large and electron hopping governs the electrical conductivity of polymer composites. Upon increasing the concentration of the nanofillers to a certain value (percolation threshold), the electrical conductivity increases remarkably due to the formation of microscale conductive networks. The electrical behavior of these nanocomposites strongly depends on the microstructure and intrinsic properties of the nanofillers in the matrix (dispersion, orientation, aspect ratio, and intrinsic conductivity of the nanomaterials).

**Figure 8:**
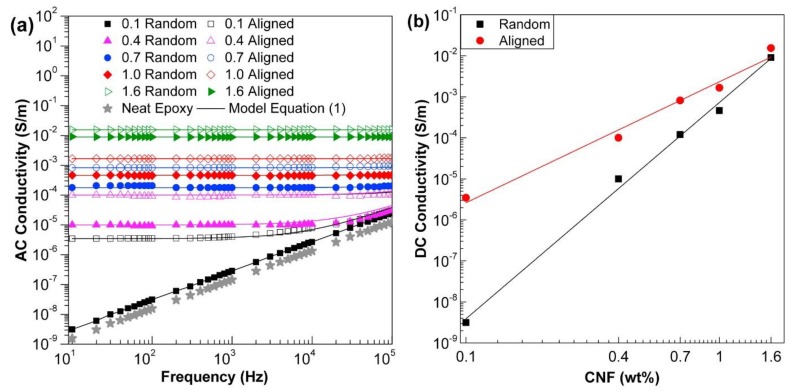
(**a**) AC and (**b**) DC electrical conductivity of the epoxy nanocomposites containing randomly-oriented or aligned CNFs in the epoxy nanocomposites [18]. Reproduced with permission. Copyright Elsevier Ltd., 2015.

The alignment of carbon nanomaterials has been shown to effectively enhance the electrical conductivities of polymer nanocomposites in the alignment direction. An increase of up to three orders of magnitude has been reported by the present authors in the electrical conductivities of epoxy nanocomposites containing electric field or magnetic field aligned CNFs or GNPs [17,18,19,20,84]. For instance, Figure 8 demonstrates the great improvement in the AC and DC electrical conductivities of epoxy nanocomposites with aligned CNFs. Additionally, anisotropic electrical conductivities have been achieved in furan resin/MWCNTs composites, prepared using the doctor blade technique [95]. The shear-induced alignment in this technique gives rise to higher electrical conductivity in the blading direction than that of a direction perpendicular to it. Another example is the epoxy composites containing graphene aerogel with a highly aligned structure prepared by the unidirectional freeze casting method, reported by Wang et al. [96]. Due to the interconnected and aligned structure of graphene aerogel, the resultant epoxy composites show exceptional anisotropic electrical conductivity and present an extremely low percolation threshold of 0.007 vol %.

**Figure 9:**
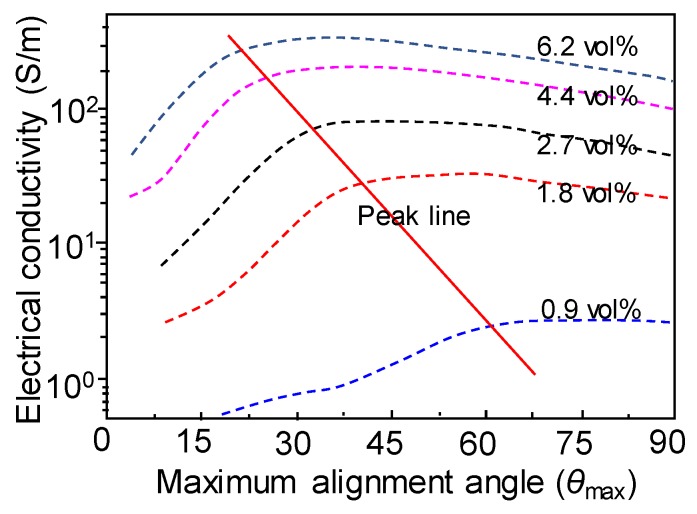
Electrical conductivity predicated by an improved 3D percolation model reported in a previous study [97], as a function of alignment direction. Data incorporated from a previous study [97].

However, theoretical modeling of the electrical conductivities of nanocomposites with aligned CNTs indicates that the maximum electrical conductivity is obtained for partially aligned CNTs rather than perfectly aligned ones (Figure 9) [97]. When CNTs are highly aligned (θ_max_ equal or close to 0), the CNTs are parallel to each other and are less likely to form interconnected conductive electron paths. Upon increasing θ_max_, a conductive network starts to form, leading to a remarkable increase in electrical conductivity. However, if there is a further increase in the θ_max_ (more random dispersion), more contact junctions and longer connective paths are formed, resulting in lower electrical conductivity. It is also interesting to note that the higher the volume fraction, the lower is the optimal orientation, as indicated by the solid line in the Figure 9. A similar conclusion was reported by Liu et al. [98], i.e., perfectly aligned MWCNTs lead to a lower conductivity while partially aligned MWCNTs with an alignment angle of approximately 30° achieved the highest electrical conductivity at a fixed volume fraction (2, 3, or 5 vol %). More recently, Xia et al. [99] presented a new effective medium theory to determine the electrical conductivity of polymer nanocomposites containing highly aligned graphene. They demonstrated that nanocomposites with highly aligned graphene show different percolation thresholds in the in-plane and out-of-plane directions. The in-plane and out-of-plane conductivities differed greatly when the inclination angle was close to zero (perfectly aligned) and approached the same value as nanocomposites with randomly oriented graphene (θ = π/2). Moreover, above the percolation threshold concentration, the difference between the in-plane and out-of-plane conductivity of aligned nanocomposites is much larger than that before percolation.

### 3.2. Thermal Properties

In addition to excellent electrical conductivity, carbon nanomaterials also show very high thermal conductivity. For instance, the theoretical thermal conductivity of an individual CNT is nearly 3000 W·m^−1^·K^−1^, while a 2D graphene sheet has a high thermal conductivity of up to around 5300 W·m^−1^·K^−1^ [100]. Therefore, they have been proposed as promising fillers to enhance the thermal conductivity of polymer materials (thermal conductivities near 0.2 W·m^−1^·K^−1^). For example, noncovalently functionalized graphene flakes greatly enhance the thermal conductivity of epoxy resin to 1.53 W·m^−1^·K^−1^ [100,101]. However, despite the tremendous efforts made to improve the thermal conductivity of polymer materials by improving dispersion and interfacial adhesion, the thermal conductivity is still far below the theoretical predictions. The reasons are not well understood and possible causes for the disparity include the high interfacial thermal resistance between nanofillers and polymers and the formation of voids in the polymer composites [102]. Both the 1D carbon materials like CNTs and CNFs and 2D carbon materials like graphene exhibit anisotropic thermal conductivity. For instance, the theoretical in-plane thermal conductivity of graphene is up to approximately 5300 W·m^−1^·K^−1^, while the thermal conductivity in the through-the-thickness direction is only around 10–20 W·m^−1^·K^−1^ [82]. The random orientation of the carbon nanomaterials prevents effective unidirectional heat transfer and thus reduces the thermal conductivity of the nanocomposites. It is highly preferred that the nanofillers are oriented uniformly throughout the composites, which would be more favorable to take advantage of the high in-plane thermal conductivity of graphene or longitudinal thermal conductivity of 1D CNTs and CNFs.

**Figure 10:**
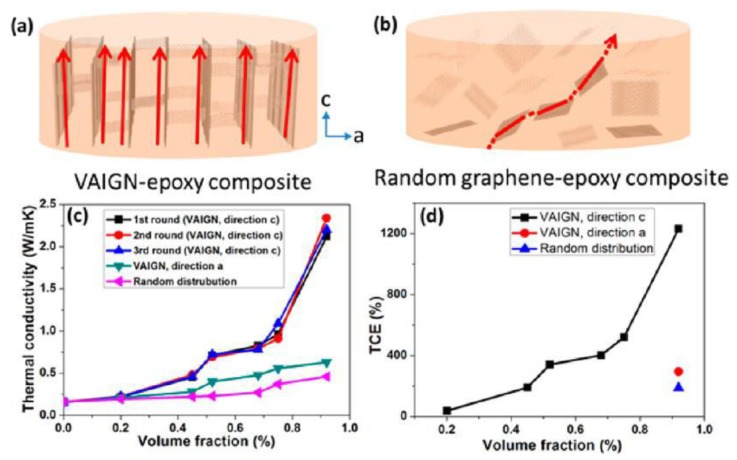
(**a**,**b**) Schematic illustration of the heat transfer in epoxy composites containing vertical graphene or randomly oriented graphene. (**c**,**d**) Thermal conductivity and thermal conductivity enhancement (TCE defined as (*K* − *K*_m_)/*K*_m_, where *K* and *K*_m_ represent the thermal conductivity of the composite and neat epoxy matrix) of the two epoxy composites [29]. Reproduced with permission. Copyright American Chemical Society, 2016.

Vertically aligned CNTs grown using CVD has shown exceptional thermal conductivity, as high as some metals such as aluminum, reaching ~265 W·m^−1^·K^−1^ [102]. When infiltrated with a thermoset epoxy (thermal conductivity of ~0.26 W·m^−1^·K^−1^), nanocomposites with a very high thermal conductivity were obtained. Vertical aligned CNTs at 17 vol % significantly enhanced the thermal conductivity of epoxy by as much as a factor of 18 in the aligned direction (4.87 W·m^−1^·K^−1^), much greater than that of the unaligned CNT composites. Similarly, vertically aligned graphene demonstrated a dramatic enhancement (1231%) in the thermal conductivity of epoxy, at an ultralow loading of 0.92 vol % (Figure 10). Due to the aligned and interconnected network, the vertical aligned rGO/epoxy composites show anisotropic thermal conductivity. There is a distinct contrast between direction *c* and *a* (as indicated in Figure 10), and the difference between these two directions increases at higher volume fractions. A significant improvement in the thermal conductivity of polymers can also be achieved by electric field [17], magnetic field [103], or melt-extrusion [104] induced alignment of graphene or CNTs. For instance, the thermal conductivity of magnetic CNTs/polyvinylidene fluoride composites aligned by magnetic field show anisotropic thermal conductivity, and in the alignment direction, a 62% increase was achieved compared to that of the unaligned composite [103].

### 3.3. Mechanical Properties

The carbon nanomaterials such as CNTs and graphene have exceptional mechanical properties, making them promising nano-reinforcements for polymers. The translation of the unique nanoscale merits of individual carbon nanomaterials to macroscale properties of polymer composites enables the development of nanocomposites with greatly improved tensile strength, stiffness, and fracture toughness. However, it remains a major challenge to take advantage of the excellent full of their mechanical potential, and the random orientation of the nanomaterials is considered as one of the possible reasons for this. Indeed, aligned carbon nanofillers have demonstrated a more significant enhancement in mechanical properties in the alignment direction compared to their counterparts with randomly-oriented carbon nanofillers [18,63,72,75,103,104,105,106,107,108]. Shi et al. reported that NiO- and CoO-coated CNFs can be well-aligned in the polymer under a moderate magnetic field (<3 T) [105], and, as a result, anisotropic tensile strength was observed. The tensile strength in the direction normal and parallel to the applied field was 12.1 and 22 MPa, respectively. Thostenson et al. developed highly aligned polystyrene nanocomposite films by extruding the polymer melt and drawing the film prior to cooling [109,110], and found that the enhancement of the elastic modulus by the aligned nanotubes was five times greater than their randomly oriented counterparts. In addition, the orientation of the nanotubes resulted in a significant improvement in the yield strength and ultimate strength, compared to the unreinforced polystyrene films. The increases in modulus and strength indicate that nanotubes act as a fiber-like reinforcement in transferring load from the matrix via shear in the aligned composites. A micromechanical approach for modeling of short fiber composites has been modified to predict the elastic modulus of nanocomposites with aligned nanotubes. The elastic modulus of the nanocomposites can be expressed as [110]:

(10) $$E_{c}=E_{m}\left(1+2\left(\frac{l}{d}\right)\left(\frac{E_{f}/E_{m}-d/4t}{E_{f}/E_{m}+l/2t}\right)V_{f}\right)\times {\left(1-\left(\frac{E_{f}/E_{m}-d/4t}{E_{f}/E_{m}+l/2t}\right)V_{f}\right)}^{-1}$$

where $E_{c}$ is the composite elastic modulus, $V_{f}$ is the CNT volume fraction, $E_{f}$ and $E_{m}$ are the modulus of CNTs and polymer matrix, l, d, and t are length, diameter, and outer layer thickness of CNTs, respectively.

Recently, Shafi et al. demonstrated that the electric field-induced alignment of MWCNTs not only gives rise to much more improved elastic modulus in the alignment direction, but also greatly improves fracture toughness compared to the randomly-oriented MWCNTs [106]. It is worth mentioning that the transverse moduli were lower than that of the composites containing randomly-oriented CNTs due to the presence of alternating resin-rich and aligned CNT bundle regions. Fractographic studies indicate that the dominant toughening mechanisms include crack tip deflection and CNT pull-out which are more significant in the composites with CNTs aligned normal to the crack growth path. In recent studies by the present authors, an AC electric field or magnetic field was used to align CNFs and graphene in epoxy to simultaneously improve the electrical conductivity and fracture toughness [17,18,19,20,84]. The alignment greatly enhances the fracture energy of the epoxy due to a higher proportion of nano-reinforcements participating in multiple toughening mechanisms, including (a) plastic void growth initiated from the microvoids created by the debonded nano-reinforcements ahead of the crack tip; (b) debonding and pull-out of the nano-reinforcements, and (c) bridging and rupture of the nano-reinforcements behind the crack tip. Therefore, the fracture energy of the epoxy nanocomposites is contributed to several by different mechanisms:

(11) $$G_{\mathrm{Ic}}=\;G_{\mathrm{CU}}+\;\Delta G_{\mathrm{pull}-\mathrm{out}}+\Delta G_{\mathrm{rupture}}+\Delta G_{\mathrm{db}}+\Delta G_{v}$$

where $G_{\mathrm{CU}}$ denotes the fracture energy of the unmodified epoxy,$\;\Delta G_{v}$, $\Delta G_{\mathrm{pull}-\mathrm{out}}$, $\Delta G_{\mathrm{rupture}}$, and $\Delta G_{\mathrm{db}}$ is the energy dissipated due to the plastic void growth mechanism, pull-out, rupture, and debonding of the nano-reinforcements. The contributions of these four major toughening mechanisms were quantitatively studied. By considering the conventional theories for fiber reinforcements, the fracture energy of epoxy nanocomposites reinforced by 1D CNFs can be calculated by [19]:

(12) $$G_{\mathrm{Ic}}=G_{\mathrm{CU}}+\frac{V_{f}\sigma_{f}^{2}d_{f}}{8\tau_{i}}+\frac{V_{f}\sigma_{f}^{2}\;l_{f}}{2\;E_{f}}+\frac{V_{f}l_{\mathrm{po}}G_{i}}{d_{f}}+{\left(1+\frac{\mu_{m}}{\sqrt{3}}\right)}^{2}\left[V_{f}\left(\frac{d_{v}^{2}}{3d_{f}^{2}}-1\right)\right]\sigma_{y}r_{\mathrm{yu}}K_{\mathrm{vm}}^{2}$$

where $l_{f}$, $d_{f}$, and $V_{f}$ are the length, diameter, and volume fraction of the nano-reinforcements, $\sigma_{f}$, $E_{f}$, and $l_{\mathrm{po}}$ are respectively the tensile strength, Young’s modulus, and pull-out length of the nano-reinforcements, $\tau_{i}$, $G_{i}$ are respectively the interfacial shear strength and interfacial fracture energy associated with debonding of the nano-reinforcements, $\mu_{m}$ is the pressure dependent yield stress constant, $K_{\mathrm{vm}}$ is the maximum stress concentration for the von Mises stresses around debonded nano-reinforcements, $\sigma_{y}$ and $r_{\mathrm{yu}}$ are the tensile yield stress and the plastic zone size at the fracture of the unmodified epoxy, respectively, and $d_{v}$ is the diameter of the voids.

Similarly, the model for estimating the fracture energy of graphene reinforced epoxy nanocomposites can be developed by assuming the voids created by debonded graphene having shape similar to an ellipsoid, the continuum mechanics are valid at the nanoscale, and the interfacial stress between the graphene and epoxy is constant. The expression to model the fracture energy is:

(13) $$G_{\mathrm{Ic}}=\;G_{\mathrm{CU}}+\;\frac{V_{f}\;t\;W\;\sigma_{f}^{2}}{2\;\left(t+W\right)\;\tau_{i}}+\;\frac{V_{f}{\;\sigma }_{f}^{2}\;l_{f}\;}{2\;E_{f}}+\;\frac{2V_{f}\;\left(t+W\right)\;l_{\mathrm{po}}\;G_{i}}{\;t\;W}+\;{\left(1+\frac{\mu_{m}}{\sqrt{3}}\right)}^{2}\left[V_{f}\left(2\pi -1\right)\right]\sigma_{y}r_{\mathrm{yu}}K_{\mathrm{vm}}^{2}$$

where W and t are the thickness and width of GNPs respectively.

**Figure 11:**
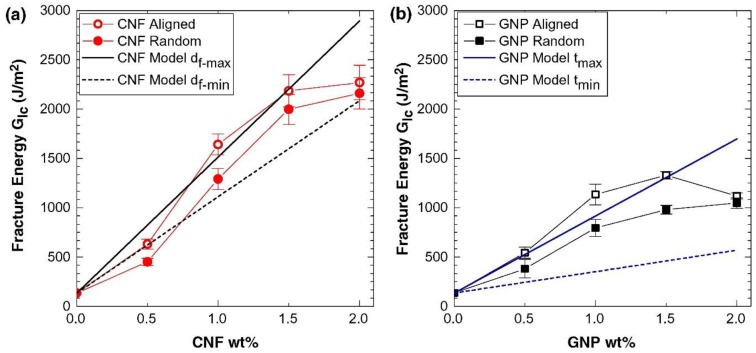
Comparison of fracture energies obtained by experiments and theoretical calculation (for the nanocomposites containing aligned or random-oriented nano-reinforcements) as a function of (**a**) CNFs and (**b**) graphene content in the epoxy nanocomposites [19]. Reproduced with permission. Copyright Elsevier Ltd., 2016.

It is worth noting that the models discussed above are based on the assumption that all carbon nanomaterials were aligned. That is, the $V_{f}$ is the effective volume fraction of carbon nanomaterials that participate in the toughening mechanisms and is assumed to be equal to the volume fraction of nanomaterials. The fracture energy calculated from the model and a comparison with the experimental results are presented in Figure 11. The experimental results agree well with the calculated fracture energies. It is found that the two most dominating toughening mechanisms are the pull-out of the nano-reinforcements and void growth. The alignment increases the number of nano-reinforcements participating in the toughening mechanism, such as plastic void growth, interfacial debonding, and pull-out of nano-reinforcements, leading to a more significant improvement in fracture energy.

## 4. Applications

### 4.1. Electromechanical Sensors

In recent years, the use of carbon nanomaterials-based polymer nanocomposites for the application of strain/pressure sensors has generated much interest. The incorporation of carbon nanomaterials with high electrical conductivity enables the development of electrically conductive polymer nanocomposites which can be used as piezoresistive or resistance-type strain/pressure sensors. The piezoresistivity of such polymer nanocomposites originates from the following mechanisms. (a) The intrinsic piezoresistivity of carbon nanomaterials; (b) significant change in the conductive networks of the carbon nanomaterials, e.g., loss of contact; (c) tunneling resistance change in neighboring carbon nanomaterials. It is widely recognized that the contribution of intrinsic piezoresistivity of CNTs to the total piezoresistivity of their polymer nanocomposites sensors may be very limited due to the poor stress transfer from the polymer matrix to the CNTs, which is caused by the large elastic mismatch between the CNTs and the polymer, and the weak interface strength [94]. The variation of the conductive networks and tunneling resistance changes are the dominant mechanisms contributing to the piezoresistivity of such sensors. Therefore, the alignment of carbon nanomaterials in polymers modifies the conductive network, resulting in significant influences on the piezoresistivity.

In 2011, Høyer and coworkers [111] demonstrated that CB (0.1 vol %) forms wire-like strings in a polymer matrix when exposed to an AC electric field, leading to greatly improved electrical conductivity and piezoresistive sensitivity with a gauge factor of ~150 when subjected to stretching in the alignment direction. In contrast, polymer thin films containing 12 vol % of nonaligned CB are conductive but not sensitive to similar stretching. In the same year, Oliva-Avilés et al. [112] reported the investigation of piezoresistivity of polymer composite films containing MWCNTs aligned by an AC electric field. The alignment of 0.75 wt % of MWCNTs improves sensitivity and linearity with the gauge factor increasing from ~0.7 to 1.49. Meanwhile, the composites containing 0.5 wt % of aligned MWCNTs show a gauge factor up to ~2.78 compared to the composites containing 0.5 wt % of randomly-oriented MWCNTs showing too low electrical conductivity to be used as piezoresistive sensors. More detailed studies on the piezoresistivity in both the transverse and parallel directions relative to the alignment of CNTs were conducted by Sengezer et al. [113], and indicate that the gauge factor in the transverse direction is 5 times more than that measured in the axial direction and that both were higher than their unaligned counterparts. However, Miao et al. [114] found that the CNT networks aligned at 90° to electrodes (parallel to stretching direction) exhibited about the same sensitivity as randomly oriented CNT networks. But, CNT networks aligned at 0° to electrodes (transverse to stretching direction) exhibited higher piezoresistive sensitivity than the randomly oriented CNT networks.

**Figure 12:**
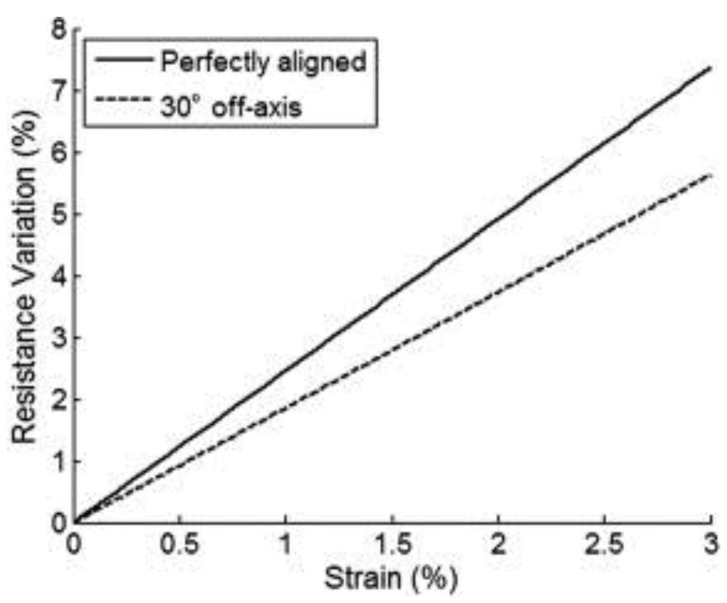
Electromechanical response for CNTs that are perfectly aligned or 30° off axis [116]. Reproduced with permission. Copyright Elsevier Ltd., 2010.

The effects of alignment of carbon nanomaterials on piezoresistivity have been qualitatively studied using multiscale modeling [115,116]. Theodosiou et al. [116] used atomistic and microscale models to numerically model the strain sensing potential of CNTs reinforced insulating polymers. The results indicated that polymer nanocomposites with oriented nanotubes, like CNT-fibers and CNT-carpets, were expected to show significantly higher sensitivity, while nanotubes aligned perpendicular to the loading direction showed no sensitivity to strain. The off-axis angles, of up to 30°, decreased the sensitivity (Figure 12) with slope (gauge factor) drops of ~30%. On the contrary, Rahman et al. [115] demonstrated that sensitivity increases as CNTs become more aligned with the cut-off angles ($\theta_{\mu }$), decreasing from 90° to 30° at low strains (<1%). But as the $\theta_{\mu }$ further reduces to 15°, the sensitivity decreases slightly. In the investigated strain range, the partially aligned CNT networks ($\theta_{\mu }$ ~ 30° or 45°) exhibited a larger resistance change under the same applied strain.

### 4.2. Structural Health Monitoring

Conductive polymer nanocomposites containing carbon nanomaterials are currently being considered as alternatives to conventional metallic strain gauges for structure health monitoring. The local damages in structures such as cracking, delamination, and fastener loosening, are often difficult to detect but have long term implications on structure performance. Such damages are caused by mechanical and environmental conditions including impact, shock loading, or extreme changes in temperatures. Nondestructive damage inspection techniques such as eddy current, X-ray, or ultrasonic are commonly used but usually require disassembly for inspection [117]. Some emerging structural health monitoring approaches include the use of strain gauges, accelerometers, piezoelectric or piezoresistive sensors, and fiber optic sensors. Many of these methods can only sense damage in areas close to the locations where the sensors are placed. The damage self-sensing capability of carbon fiber-reinforced composites has been investigated [118], which demonstrates that high electrical conductivity along the fiber direction can be used for in situ monitoring of strain and damage through electrical resistance measurements. However, this approach does not provide much insight into matrix-dominated fractures and is not applicable to composites where fibers are not conductive, such as glass fibers or aramid fibers.

Recently, Thostenson and co-workers [119,120] demonstrated that conducting CNT networks formed in an epoxy matrix can be utilized to detect the onset and progression of damages in glass fiber reinforced epoxy composites by direct current measurements. By introducing electrically conductive carbon nanomaterials into an insulating polymer matrix, it is possible to detect various matrix-dominated early stage failure modes. This approach does not need additional equipment and largely improves the efficiency of composites structures for industries such as aerospace and construction. However, to achieve good electrical conductivity, it is necessary to add a large content of carbon nanofillers (higher than percolation threshold), which significantly increases the viscosity. The increased viscosity of the resin mixtures causes difficulties in composite manufacturing processes such as resin infusion. Therefore, lower concentrations of nanomaterials are desirable. The enhanced electrical conductivity by alignment will give the composites new sensing capability. The present authors [91] recently investigated the crack-detection capability of multiscale glass fiber/epoxy composites containing CNFs aligned in the through-the-thickness direction by an AC electric field. In situ crack propagation and internal damage sensing capability were investigated by detecting crack growth during mode I interlaminar fracture tests. It is interesting to note that the alignment of CNFs, in the transverse direction to that of the crack growth, not only enhanced the mode I fracture toughness, but also improved the damage-sensing capacity. Based on a computational study, the greatly increased through-the-thickness electrical conductivity contributes to the enhanced damage-sensing performance.

### 4.3. Electromagnetic Interference (EMI) Shielding

Due to the rapid development of telecommunications and electronics, how to effectively shield or block EMI is becoming a serious concern for modern society. Conventionally, metals are usually used in this regard due to their high electrical conductivity. However, because they are very heavy and prone to corrosion in harsh environments, it is necessary to develop new shielding materials. The main mechanisms of EMI shielding are the reflection and absorption of electromagnetic radiation. To isolate equipment from EM radiation, the shielding materials must have mobile charge carriers, i.e., they should be electrically conductive. Therefore, flexible, electrically conductive lightweight polymer composites are attracting considerable attention for the application of EMI shielding. The EMI shielding effectiveness of a composite material is dependent mainly on its electrical conductivity, dielectric constant, and the aspect ratio of the fillers [121]. Recently, carbon nanomaterials have gained popularity as fillers to develop polymer nanocomposites for EMI shielding thanks to their excellent electrical conductivity [122]. To fully utilize the potential of carbon nanomaterials, rational assembly within the polymer matrix is necessary. As discussed in Section 3.1, the alignment of carbon nanomaterials greatly increases the electrical conductivity in the alignment direction. It is thus expected that the polymer nanocomposites with fillers aligned in a preferential direction may exhibit better EMI shielding performance.

**Figure 13:**
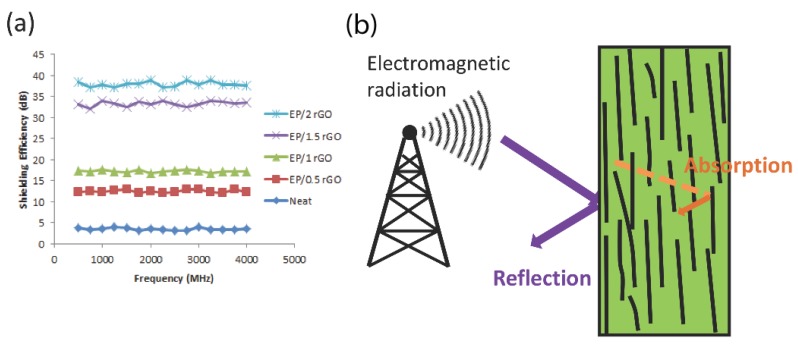
EMI SEs of (**a**) rGO/epoxy and (**b**) schematic of electromagnetic wave shielding through the thickness of composites [121]. Reproduced with permission. Copyright John Wiley & Sons, Inc., 2014.

Yousefi and coworkers [121] developed self-aligned rGO/polymer nanocomposites via the aqueous casting method. Their work indicated that rGO/epoxy composites exhibit morphology evolution from an isotropic to a layered structure upon increasing graphene content. The morphology evolution was confirmed by the results that identical electrical conductivity is obtained between in-pane and out-of-plane up to ~1 wt % of rGO, while significant anisotropic conductivity is observed for composites containing more than 1 wt % of rGO. The alignment of the graphene also results in composites with higher dielectric constants. The EMI shielding efficiency (SE) of the rGO/epoxy nanocomposites are presented in Figure 13a, which is among the highest value reported in the literature for carbon filler/polymer composites containing such low loading of fillers. The rGO sheets aligned in the in-plane direction contribute to the shielding of incident electromagnetic (Figure 13b). Similarly, self-aligned rGO/poly (vinylidene-hexafluoropropylene) thin films showing SE up to 30 dB were fabricated by Kumar et al. [123]. Their research provided useful insight into the development of polymer nanocomposites with high EMI shielding effectiveness.

### 4.4. Damage-Tolerant Structures

Advanced light-weight FRPs have been increasingly utilized in aerospace, aviation, automotive, and construction industries due to their excellent mass-specific mechanical properties. Fiber reinforced polymer composites, especially carbon fiber reinforced polymer composites (CFRPs), are widely used, either for primary structures or for secondary structures. For instance, CFRPs have been utilized for aircraft fuselages and wing structures, helicopter rotors, bridges, and large civil infrastructures. Such composites show outstanding in-plane properties including high strength and stiffness. In contrast, the interlaminar region is relatively weak with a low interlaminar strength and fracture toughness. The poor out-of-plane properties make them susceptible to microscale damages such as delamination and interfacial debonding. The use of carbon nanomaterials offers tremendous potential to improve the properties of FRPs. As discussed in Section 3.3, CNFs and graphene can significantly improve the fracture toughness of epoxy and the main reinforcing mechanisms include plastic void growth of the matrix, debonding, pull-out, and fracture of the nanofillers, and crack bridging. Moreover, the use of CNTs in composite structures has been proven to enhance the impact strength and fatigue life of composites, resulting in more damage tolerant structures.

In particular, several recent studies have demonstrated that the alignment of carbon nanomaterials in the through-the-thickness direction further improves the out-of-plane properties of the composites [91,124,125,126,127]. For instance, the present authors recently demonstrated that the alignment of CNFs, normal to the crack growth direction, greatly increases the interlaminar fracture toughness by ~100%, compared to the unmodified glass fiber reinforced composites, while the randomly-oriented CNFs yield only a ~50% improvement [91]. Wicks et al. [124] reported the fabrication of “fuzzy fiber” reinforced polymer laminates where aligned CNTs were grown on advanced microfibers in a woven ply. The addition of aligned CNTs has been proven to greatly enhance the steady-state Mode I fracture toughness of laminated composites, dependent on the chosen fiber and matrix type as well as CNT length.

In summary, the use of carbon nanomaterials as fillers has been proven to be able to improve the damage tolerance of FRPs. The alignment of these fillers in the through-the-thickness direction can improve the relatively weak interlaminar properties and reduce the occurrence of detrimental damages, and thereby, prolong the service life of structural composites.

## 5. Challenges and Opportunities

Commercial and research interest in polymer composites with carbon nanomaterials are rapidly increasing and the continuous effort of improving the multifunctional properties of polymer nanocomposites have resulted in the development of various alignment techniques. In this review, we have provided an extensive overview of the currently available approaches to align carbon nanomaterials in a polymer matrix to develop functional nanocomposites. The physical mechanisms of the electric field and magnetic field-induced alignment have been thoroughly discussed. The unique properties created by the alignment of carbon nanomaterials and potential applications are also discussed in this review. The alignment of the carbon nanomaterials in a polymer matrix can be achieved during (in situ) or before (ex situ) the formation of composite. The preferential assembly and alignment drastically improves the electrical, thermal conductivity, and mechanical properties. The enhanced electrical conductivity promotes the use as electromechanical sensors with higher sensitivity than their unaligned counterparts. Moreover, the high electrical conductivity induced by the alignment brings them closer to practical applications in structural health monitoring and EMI shielding. When nanofillers are aligned in the through-the-thickness direction of fiber reinforced polymer composites, the relatively weak interlaminar properties are greatly enhanced, leading to much more damage tolerant structures. These outstanding properties, due to the alignment of nanofillers, provide these polymer nanocomposites with promising potential for use in aerospace, automotive, electronics, and medical equipment.

Although there are numerous techniques for directionally aligning carbon nanomaterials, it is worth noting that challenges remain, and future development opportunities include:

1. Each of the established alignment techniques have both advantages and disadvantages, as discussed in the early sections of this review and summarized in a previous study [23]. For instance, as discussed in Section 2.1.1, polymer infiltration process may cause structural damage to the vertical aligned CNTs and the polymer must be highly compatible with CNTs to ensure the complete infusion. Other techniques based on shear force, mechanical stretching, or electrospinning may damage the structure of carbon nanomaterials. For alignment by external fields (electric field or magnetic field), high field strength is usually required for preparing bulk composites. Due to the relatively low magnetic susceptibility, carbon nanomaterials may need functionalization by introducing magnetic particles so that they can be aligned under a low-strength magnetic field.
2. Large scale production remains a great challenge. Cakmak et al. [48] recently developed a large scale manufacturing platform to align nanoparticles in the through-the-thickness direction by a new roll-to-toll processing line. Meanwhile, to prepare a large-scale nanocomposite film with nanofillers aligning in the in-plane direction, Sun and coworkers [128] proposed an array of parallel wire electrodes instead of a single pair of parallel plate electrodes. Apart from these attempts, very few reports could be located in the literature on the translation of lab-scale alignment techniques to practical applications for processing large size nanocomposites. Therefore, further studies and investigations are needed to scale-up the alignment process.
3. The effects of alignment on some properties such as piezoresistive sensitivity of polymer nanocomposites are not conclusive and further detailed investigations, both experimentally and/or numerically, are needed. It is anticipated that significant improvement in the design and fabrication of electromechanical sensors is possible by manipulating the orientation of nanomaterials.
4. The type of polymer matrix investigated to date is limited and more studies are needed to understand how alignment will affect the properties of other polymer matrix composites. Moreover, very few studies have been reported on the alignment of hybrid nanofillers. The different structures and properties of the nanofillers may result in multiscale aligned structures, which will be of great interest for designing new functional nanocomposites.
5. The different alignment techniques may be combined to create new composites materials. For instance, shear forced-induced alignment during 3D printing of polymer nanocomposites may be combined with other techniques (e.g., applying electric or magnetic fields) to develop nanocomposites with well-aligned microstructures.
